# Establishing a new animal model for muscle regeneration studies

**DOI:** 10.22099/mbrc.2019.34611.1433

**Published:** 2019-12

**Authors:** Hossein Pourghadamyari, Mohammad Rezaei, Ali Ipakchi-Azimi, Shahram Eisa-Beygi, Mohsen Basiri, Yaser Tahamtani, Hossein Baharvand

**Affiliations:** 1 Student Research Committee, Kerman University of Medical Sciences, Kerman, Iran; 2 Department of Clinical Biochemistry, Afzalipour School of Medicine, Kerman University of Medical Sciences, Iran; 3 Department of Stem Cells and Developmental Biology, Cell Science Research Center, Royan Institute for Stem Cell Biology and Technology, ACECR, Tehran, Iran; 4 Department of Medical Laboratory Science, Medical Science Faculty Babol Islamic Azad University, Babol, Iran; 5 Department of Radiology, Medical College of Wisconsin, Milwaukee, USA; 6 Department of Developmental Biology, University of Science and Culture, Tehran, Iran

**Keywords:** Transgenic animal model, Muscle regeneration, Tol2 transposase, Zebrafish

## Abstract

Skeletal muscle injuries are one of the most common problems in the worldwide which impose a substantial financial burden to the health care system. Accordingly, it widely accepted that muscle regeneration is a promising approach that can be used to treat muscle injury patients. However, the underlying mechanisms of muscle regeneration have yet to be elucidated. The muscle structure and muscle-related gene expression are highly conserved between human and zebrafish. Therefore, the zebrafish can be considered as an ideal animal model in muscle regeneration studies. In this study, Tol2 transposase was applied to produce Tg(mylpfa: cfp-nfsB) zebrafish model that express a fusion protein composed of cyan fluorescent protein (CFP) and nitrorudactase (NTR) under control of mylpfa promoter. The results showed that MTZ (Metronidazole) treatment of Tg(mylpfa:cfp-nfsB) zebrafish larvae can lead to muscle injury by selective ablation of muscle cells. And also, results confirmed the muscle regeneration ability of the transgenic larvae after withdrawal of Mtz for three days. Overall, The results of this study suggest that the Tg(mylpfa:cfp-nfsB) zebrafish model can be used in muscle regeneration study in order to elucidate the mechanisms of this process.

## INTRODUCTION

Skeletal muscle impairment is one of the most common disabilities worldwide which are usually caused by aging [1], disease such as muscle dystrophy [2], myotoxic agents [3], trauma and ischemia [4]. Accordingly, muscle regeneration is a growing research area which has shown promising results for muscle injury treatment [5]. 

The inverse action of the mechanisms involved in the pathogenesis of diseases can be considered as a novel strategy in treatment of some diseases [6]. Application of animal models in biomedical researches help researchers to identify new solutions for treatment or diagnosis of the diseases. Throughout the years various animal disease models have been being used by researchers to explore the underlying molecular and cellular mechanisms of the diseases [7-9].

A number of studies have demonstrated that *Pax7* transcription factor as muscle tissue stem cells (satellite cells) marker has a critical role in muscle development and regeneration. Studies also showed that the satellite cells are quiescent in physiological muscle conditions while in muscle injury conditions, satellite cells are activated, and along with muscle myogenic regulatory factors (MRFs) such as myod1 (myogenic differentiation 1) and myf5, play an important role in the regeneration and healing of the damaged muscles [10-12]. However, a number of key questions remain unanswered in muscle regeneration process.

One promising avenue is to produce an appropriate animal model to clarify molecular and cellular events that contribute to pathogenesis of diseases. So far, various experimental strategy such as chemical myotoxin injections [13, 14], myectomy surgery [15] and cryoinjury [16, 17] have been used in order to produce animal muscle regeneration models. However, all of these strategies are invasive, unspecific and time consuming. Thus, with respect to these drawbacks, use of transgenic animal models seems to be more reasonable. 

Zebrafish *(Danio rerio)* has recently attracted researcher’s attention as an animal model [18-20]. As an eukaryotic model, zebrafish model is characterized by its features include: 1) small size and robustness, 2) production of large number offspring, 3) whole genome sequencing, 4) transparency at early developmental stages, 5) simple organ structure, 6) having regenerative capacity in most organs, 7) extremely fast growing and development, 8) similar genetic structure to humans 

Furthermore, it is worth noting that, the muscle structure, myogenesis and gene expression are highly conserved between zebrafish and human [21]. Recently, Li et al. published a paper in which they reviewed Skeletal Muscle Properties in Zebrafish. They demonstrated molecular and cellular mechanisms of the myogenesis process in zebrafish and they also explained the advantages of Zebrafish muscle disease models for modeling human muscle disorders [22].

Regarding the aforementioned zebrafish advantages, the aim of this study was produced a transgenic zebrafish as an animal model of muscle regeneration. This transgene model harbors a nfsB prokaryotic gene, encoding nitroreductase (*NTR*) enzymes so that they are able to metabolize polynitroaromatic compounds. Previous studies have shown that the metronidasol (MTZ), one of *NTR* substrates, can be metabolized and converted into toxic substances by NTR which leads to cellular apoptosis [18, 19, 23, 24]. In this study *Tg(mylpfa: cfp-nfsB)* was produced which NTR and CFP are expressed under the control of the *mylpfa* promoter. 

## MATERIALS AND METHODS

**Genomic PCR** **: **Genomic DNA was extracted from fin samples of the adult zebrafish using Qiagen DNA extraction Kit, according to the manufacturer’s instructions (Qiagen, Germany). All components of PCR were provided in a final volume of 12.2µl under standard conditions. Then, 38 µl autoclaved, distilled Water was added to the contents of each microtube. Each microtube contained 0.2 µM each one of primers, 0.8mM of dNTPs (Invitrogen, USA), 2mM of MgCl_2_, and 0.2 µl of Platinum® Taq DNA Polymerase High Fidelity (Invitrogen, USA). Finally, 200 ng of genomic DNA was added. The PCR reaction was carried out in a Thermal Cycler (Eppendorf, Germany), with the following program: 5min at 95˚C followed by 30 cycles of 45 s at 95˚C, 50 s at 60˚C, 45 s at 68˚C, followed by with 5min at 72˚C as final extension.

**Designing and cloning genetic constructs:** For designing and cloning molecular construct, we used PCR cloning method to insert zebrafish myosin promoter (gene ID: 30429, mylpfa) into Royan Tol2 CFP2A plasmid at upstream of CFP. To this end, primers were designed to amplify 2000 bp upstream of the mylpfa coding sequence (CDS), and SphI and SalI restriction sites were flanked to 5ʹof forward and reverse primers respectively (Table 1). The next PCR cloning was conducted to insert nfsB coding sequence (gene ID: 945778) downstream of the CFP of Tol2 CFP plasmid. NdeI and EcoRI restriction sites were flanked to 5ʹ of forward and reverse primers respectively (Table 1).

**Table 1:** Primers sequence

**Primer name**	Primer sequence*
*mylpfa* forward primer	CAAC**GCATGC**AGAGGAATGAGCCACCAACTC
*mylpfa* reverse primer	TATA**GTCGAC**ACGGTATGTGTGAAGTCTAAG
*nfsB* forward primer	TAGC**GTCGAC**ATGGATATCATTTCTGT
*nfsB* reverse primer	TAGC**GGATCC**CACTTCGGTTAAGGTGATGT
*Pax7* forward primer	GCCTCTTCCGTTAGCTCCATT
*Pax7* reverse primer	TATCCCCGAGAATCCCGTCA
*myod* 1 forward primer	GCTTCCAGTCCGAGATCCAA
*myod* 1 reverse primer	AGCTGTTCCGTCTTCTCGTC
*myf5* forward primer	TCCAGTACATCGAGAGCCTTC
*myf5* reverse primer	GGCCATACAGGACTGTTGCAG
*eef1a1l1* forward primer	TACCCTCCTCTTGGTCGCTT
*eef1a1l1* reverse primer	GAAGAACACGCCGCAACCTT

* Restriction sites are determined by underline

**Transposase in vitro transcription (IVT):** At first step of in vitro Transcription, pCS2-transposase plasmid (gifted by Dr. Ekker, Ottawa, Canada) was linearized with NotI restriction enzyme and in the next step ethanol precipitation was done to remove proteins that may influence IVT reaction. mMESSAGE mMACHINE SP6 Transcription Kit (Life Technologies, USA) was used for in vitro transcription of transposase. According to the manufacturer's instructions. Briefly, 4 µl nuclease-free water, 2µl 10X buffer, 10µl 2X NTP/CAP, 1 µg linearized plasmid and 2 µl SP6 enzyme was added to reaction tube respectively and incubated at 37°C for 3 hours. DNase treatment was done to eliminate the plasmid DNA followed by Lithium Chloride (LiCl) precipitation to remove unincorporated nucleotides and proteins. The quality of mRNA product was analyzed by agarose gel electrophoresis. 

**Zebrafish maintenance:** Zebrafish were raised according to standard conditions in circulating system that help to filter waste water and also kept clean and fresh the environmental water. Zebrafish were kept at 28°C and lighting condition was 14 hrs light and 10 hrs darkness. In order to get zebrafish zygotes, desired female and male zebrafishes were put into breeding tanks and separated by a divider. The ratio of female to male zebrafish in each breeding tank was 2: 1. The tanks were left in the dark at 28°C overnight. On the next morning, the divider was removed and 10 minutes after spawning, embryos were collected and washed. The good-quality embryos were selected for genetic constructs microinjection. All animal protocols were approved by the Institutional Animal Care and Use Committee of Royan Institute. 

**Metronidazole preparation and treatment:** Metronidazole (MTZ, Sigma) was dissolved in 0.1% DMSO in E3 medium with vigorous agitation. To ablate muscle cells, the *Tg(mylpfa: cf*p*-nfsB*) larvae at 3 days post-fertilization (dpf) were placed in six-well plates containing 5 ml of 20 mM MTZ-0.1% DMSO E3 medium for 48 hours at 28.5°C in the dark. After 48 hours MTZ treatment, the larvae washed three times with E3 medium. Then, 5 ml E3 medium added to each wells of larvae and muscle regeneration analysis was carried out at 8 dpf.

**Muscle injury and muscle regeneration analysis:** To test embryos movement ability we used mechanical stimulation (shaking the embryos dish) at 5dpf and 8dpf. Here after gently shake of embryos dish, the motility was observed and recorded by stereomicroscope. Furthermore, we acquired the images at identical settings in 5pdf (after MTZ treatment) and 8dpf (3 days after MTZ washed out) using an Olympus microscope with CFP filter (Olympus, Japan). The images analyzed by ImageJ software (National Institutes of Health, Bethesda, MD, USA). The average CFP intensity of images in MTZ and non- MTZ treatment embryos was quantified as mean pixel density as described previously (25). The pixel density data were normalized against the average CFP intensity of the non-MTZ treatment embryos.

**Reverse transcription quantitative PCR (RTq PCR):** Twenty embryos were harvested at 3,5 and 8 dpf and total RNA was extracted using TRIzol (Sigma, USA) followed by DNAse treatment using RNase-Free DNase (Fermentas, Germany)were carried out according to the manufacturer’s instructions. The amount and quality of RNA were assessed by spectrophotom-etry (at 260 nm) and agarose gel electrophoresis. Total RNA (1 µg) was reverse transcribed using reverse transcriptase enzyme (Qiagene) and random hexamer primers. Real-time PCR was performed on a RotorGene 3000 Instrument (Corbett Research, Australia). mylpfa, myf5 and Pax7 expression levels were measured by specific primers. The data were normalized against eef1a1l1 transcript level and analyzed by delta delta Ct method.

**Statistical analysis:** The data are presented as mean±SD of at least three independent experiments. The statistical analyses were carried out using SPSS 13.0 (SPSS, Chicago, IL). Comparisons among all groups were performed with one-way analysis of variances (ANOVA). If significant differences were found, a post-hoc Bonferroni test was carried out. 

## RESULTS

**Figure 1:**
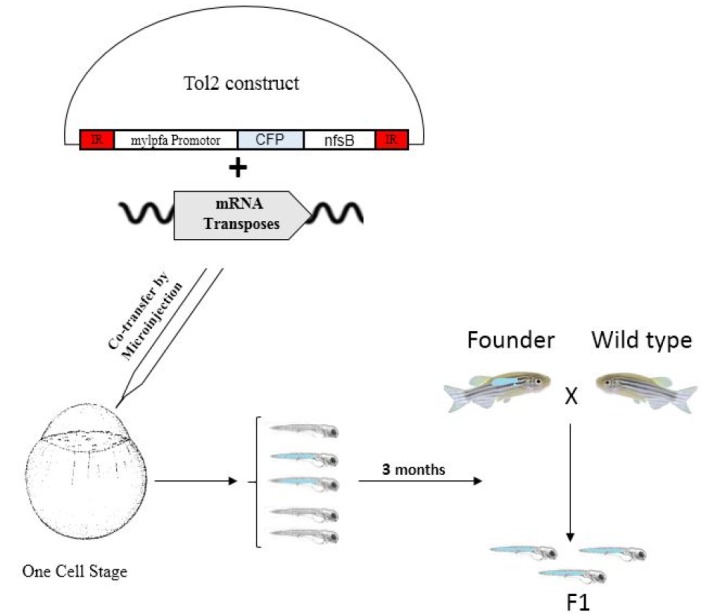
The workflow of the study. Zebrafish embryos were microinjected at one-cell-stage with the desired gene construct. Two days after injection, embryos were evaluated for CFP expression. CFP positive embryos were selected to raise as F0 embryos transgenic fish. CFP positive embryos were selected and raised. CFP positive adult zebrafishes were crossed with wild type (TU strain) to produce the F1 generation.

The results of digestion and sequencing analysis confirmed the insertion of *mylpfa* promoter and *nfsB* sequence into Tol2 plasmid. And also, gel agarose electrophoresis confirmed the quality of in vitro transcription of transposase mRNA. The workflow of this study was depicted in Figure 1. Zebrafish embryos were microinjected at one-cell-stage with the desired gene construct. Two days after injection, embryos were evaluated for CFP expression. CFP positive embryos were selected to raise as F0 embryos transgenic fish**. **Transposase mRNA and recombinant Tol2 CFP NTR were injected to 148 one-cell-stage embryos. Microscopic evaluation showed that 12 embryos were CFP positive (Fig. 1).

**Figure 2:**
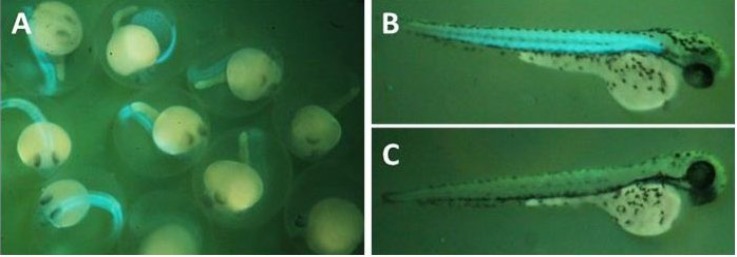
The CFP of Tg(mylpfa:cfp-nfsB) is specifically expressed in muscle cells. **A.** Tg(mylpfa:cfp-ntr) and wild type larvae 36 hrs after Tol2 construct injection. **B.** 3dpf of Tg(mylpfa:cfp-nfsB) larvae. **C.** 3dpf of and wild type larvae. (dpf (Days Post Fertilization), CFP (cyan fluorescent protein).

**Figure 3:**
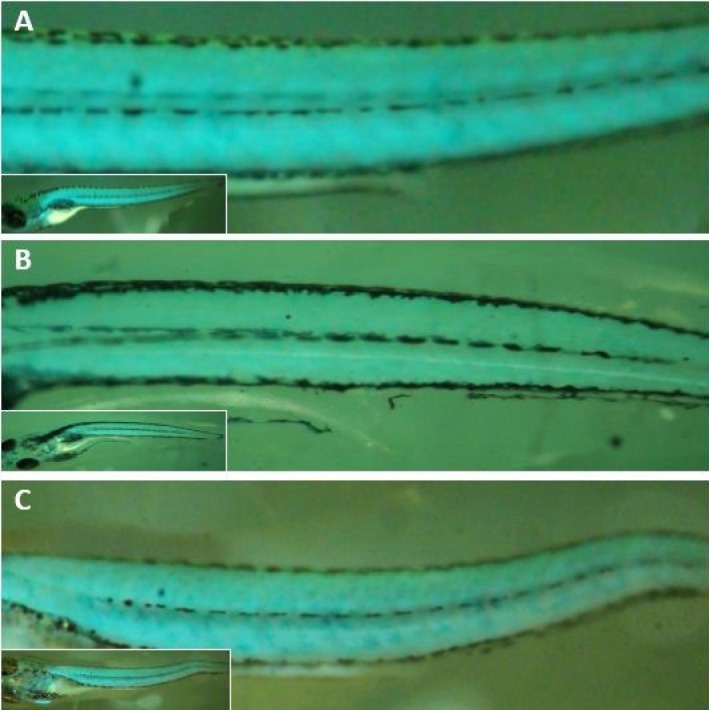
MTZ treatment specifically depletes muscle cells of Tg(mylpfa:cfp-ntr) and muscle regeneration. **A.** Before MTZ treatment (3dpf). **B.** After 48 hrs MTZ treatment (5dpf). **C.** Expression of CFP appeared again after 3-day MTZ withdrawal in larva from the MTZ treatment group (8dpf). (dpf (Days Post Fertilization).

CFP positive embryos were selected and raised (Fig. 2). CFP positive adult zebrafishes were crossed with wild type (TU strain) to produce the F1 generation (Fig.1). Microscopic evaluation reiterates to detect CFP positive F1 embryos. Of 12 CFP positive embryos, three embryos showed germline transmission. The muscle cells are targeted for ablation from 3 to 5 dpf by using muscle cell-specific expression of nitroreductase, which converts MTZ into a cytotoxic product. After 3 days of recovery (8 dpf), muscle cell ablation can be seen in decrease expression of CFP and motility of transgenic larvae (Fig. 3). It was shown that the nfsB encoded NTR can induce drug dependent apoptosis in these cells.

In order to determine whether the *Tg(mylpfa:cfp-nfsB)* transgenic fish can be used as a model of muscle regeneration, the intensity of CFP and motility of larvae after 3 days recovery were evaluated using fluorescent stereomicroscope photos and mechanical stimulation. After 3 days of recovery, from 5-8 dpf, muscle cell regeneration can be seen as increased expression of CFP and ameliorated motility of transgenic larvae (Fig. 3). In order to check the expression of previously reported marker genes for muscle degeneration and regeneration, the expression of *Pax7*, *myod1*, and *myf5* were assessed in muscle ablation and recovery conditions. We performed the expression analysis of selected target genes using RTq PCR. Although could not detect any significant change in Pax7 and myf5 expression, the trend of data suggested a slightly increased expression of *Pax7* and *myf5* before and after MTZ treatment. Our data also revealed a significant increase in the expression of *myod1* in regeneration stage (8dpf) than ablation stage (Fig. 4).

**Figuer 4:**
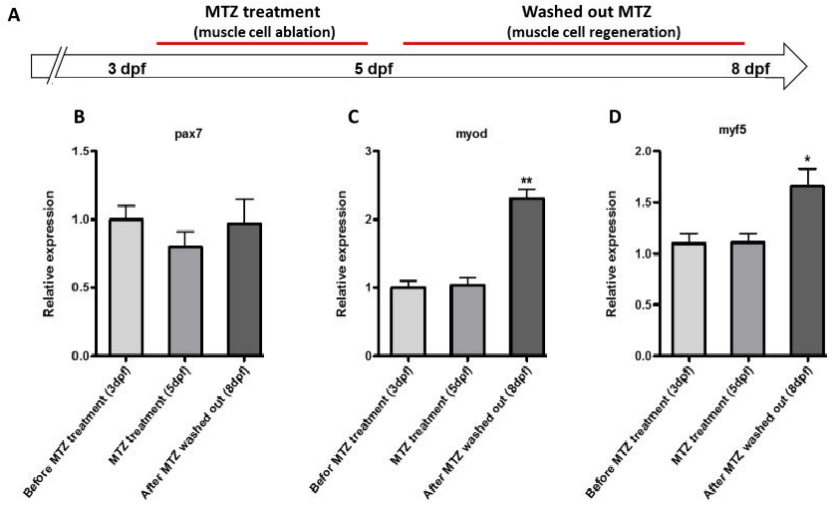
Evalute the expression of myod, myf5, and pax7 in muscle regeneration process. **A.** Schematic diagram for cell-labeling and assessment of muscle cell regeneration. **B.** Pax7 relative expression at 3 dpf, 5 dpf and 8 dpf. **C.** myod relative expression at 3 dpf, 5 dpf and 8 dpf. **D.** myf5 relative expression at 3 dpf, 5 dpf and 8 dpf. mRNA levels were quantified normalized relative to eef1a1l1 mRNA expression. *P<0.05 and **P<0.01.

## DISCUSSION

The advancement of transgenic animal models pave the way for the modeling human diseases. It is widely accepted that appropriate animal model help to identify molecular and cellular mechanisms contributing to pathogenesis of diseases. Muscle structure, synthesis and gene expression patterns are highly conserved between zebrafish and human. According to high prevalence of muscle injuries and unknown mechanisms underlying muscle regeneration, the aim of this study was to produce a transgenic zebrafish model that can use in muscle regeneration studies. 

In this study, Tol2 transposon plasmid and transposase mRNA have been injected into 148 one cell stage embryos, among them just 3 embryos showed germ line transmission of the transgene. Therefore, the efficiency of Tol2 transposase method in this study was about 1.5 percent. While other surveys such as that conducted by Kawakami [26] and Kwan [27] have reported that Tol2 efficiency system more than 50 percent. This difference can be duo to difference in the location of the injection as in this study we injected the constructs into yolk of zygotes. It seems that the difference efficiency of our result with other studies explained by Rembold et al., in 2006. They reported that injection gene constructs in yolk can be lower efficiency of Tol2 system [28].

To test the proper expression and efficiency of NTR in ablating myocytes , MTZ was added to the aquatic environment of the transgenic zebrafish. We used a mechanical stimulation for the analysis of muscle function in zebrafish. Data showed that the motility and escape response decreased after 48 hrs MTZ treatment. These findings are consistent with those of Smith et al. (2013) who showed that skeletal muscle defects in larval zebrafish decrease motility and escape response [29]. In addition, results showed that optimal concentration of MTZ was 20 mM and high concentration of MTZ (more than 20 mM) had lethal effect. 

**Figure 5:**
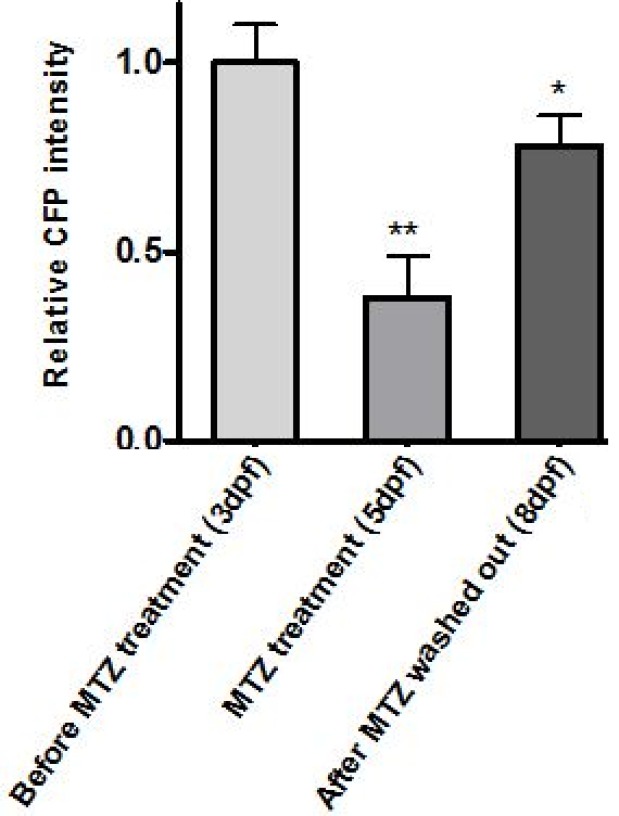
Graph of relative CFP intensities at 3 dpf, 5 dpf and 8dpf. *P<0.05 and **P<0.01, n = 6. (dpf=Days Post Fertilization).

The CFP Intensity was analyzed by fluorescent microscopic method and ImageJ software (Fig. 5). Data showed that intensity of CFP decreased in MTZ treatment embryos compared to non-MTZ treatment embryos [25]. Collectively, these data suggests that NTR are able to induce muscle destruction in the *Tg(mylpfa:cfp-nfsB)* zebrafish model.

Next, the result of mechanical stimulation test after 3 days washing out of the MTZ showed Increase of the motility and escape response of the zebrafish model. These finding indicated the potentiation of muscle regeneration in the *Tg(mylpfa:cfp-nfsB)* zebrafish model (movies). Furthermore, RTqPCR was performed to analyze expression of *myod1* and *myf5* as muscle myogenic regulatory factors and also *pax7* as a marker of satellite cells (muscle stem cells). Gene expression data analysis (Fig. 4) showed that expression of myod, myf5 and pax7 increased after 48 hrs MTZ treatment. Several previous studies indicated that one of the main event in muscle regeneration is over expression of muscle regular factors and *pax7 *[10-12]. Previous study showed that the Pax7 positive myocytes display differential reactions to muscle damage depending on developmental stage and the extent of the injury [11]. However, it seems that mtz injury is able to decrease Pax-7 expression. As the mtz as cell specific toxins was removed, Pax-7 expression starts to increase gradually.

In 2013, Otten and his colleagues introduced a new skeletal muscle regeneration model. The established model represented a local muscle injury by micropoint laser to study skeletal muscle regeneration in the zebrafish embryos [30]. It seems that this laser-mediated cell ablation model is suitable for localized skeletal muscle regeneration study. It should be noted that this method requires an advanced laser system for localized physical muscle injury. In our model metronidazole (mtz) can induce apoptosis process especially in myocytes, because Myosin promoter was used as a tissue-specific promoter to target muscle tissue and CFP and nfsB (NTR) gens would be expressed under control of this promoter. Furthermore, the specificity of the NTR/mtz system and absence of its bystander effect have been confirmed in previous study , [23, 24]. Therefore, additions of mtz as cell specific toxins lead to the precise timing of muscle injury. Severity of muscle damage also can be control by mtz concentration. And also, it must be mentioned NTR/mtz system is not an offensive method and approved by the Laboratory Animal Ethics Committees [23, 24]. Moreover, confocal microscopy can be applied to assess of muscle damage and regeneration in more detail.

However, fluorescent microscope can be used for monitoring of the muscle damage and regeneration with evaluation of CFP intensity. Notably, increasing and decreasing fluorescent intensity are represented the muscle damage and regeneration process, respectively. 

In other hands, all physical injury models are invasive and we cannot control intensity of injuries and also, they show bystander effect (adjacent tissues are damaged).

In *Tg(mylpfa:cf*p*-nfsB*) zebrafish, CFP expression is driven by the zebrafish myosin promoter, so myosin-expressing cells of the muscle were visualized. Therefore, muscle injury and muscle regeneration of Tg(mylpfa: cfp-nfsB) zebrafish were analyzed indirectly. Further immunohistological investigations using zebrafish myosin specific antibody may provide more direct information about the extent and histological details of muscular ablation and regeneration in this transgenic model.

Taken together, these results suggest that the CFP-NTR fusion protein was expressed in muscle cells of the Tg(mylpfa: cfp-nfsB) zebrafish model. Muscle cells can be specifically targeted and depleted with NTR-MTZ technology in the *Tg(mylpfa: cfp-nfsB)* zebrafish model. In addition, our data confirmed the potentiation of muscle regeneration in this model. Overall, The results of this study suggest that the *Tg(mylpfa:cfp-nfsB)* zebrafish model can be used in muscle regeneration study in order to elucidate the mechanisms of this process.
